# Current status and challenges of therapeutic targets, novel drugs and delivery systems for hepatitis B: how far to our goal?

**DOI:** 10.3389/fcimb.2025.1692924

**Published:** 2026-01-14

**Authors:** Yanmei Liao, Fei Lv, Mei Zhou, Jie Shen, Tianwen Quan

**Affiliations:** Department of Pharmacy, Public Health Clinical Center of Chengdu, Chengdu, Sichuan, China

**Keywords:** hepatitis B virus, host-targeted therapy, combination therapy, drug delivery systems, functional cure

## Abstract

Hepatitis B (HB) remains a global public health challenge, imposing significant burdens on patients and society. Therapeutic strategies and novel drug development for HB continue to be a major research focus, yet current treatments fail to achieve satisfactory clinical cure rates. To address this critical gap, more effective therapeutic approaches are urgently needed. A comprehensive understanding of the hepatitis B virus (HBV) life cycle and the immunopathogenesis of persistent HBV infection, combined with innovations in drug development and delivery systems, will lead to novel strategies for treating chronic HBV infection. This review summarizes recent advances in HBV therapeutic targets, encompassing both viral life cycle and host-directed targets. We critically evaluate emerging therapeutics, including synthetic compounds, herbal medicines, and immunomodulators, along with their supporting preclinical and clinical evidence, as well as progress in drug delivery systems including liver-targeted nanoparticles, and synergistic therapeutic strategies that combine conventional and Chinese-Western medical approaches for enhanced efficacy. Through this comprehensive analysis, this review aims to provide valuable insights for clinical management of HBV and development of innovative therapies, thereby advancing the HBV treatment field. We anticipate achieving complete cure for HB in the foreseeable future.

## Graphical Abstract

**Figure:**
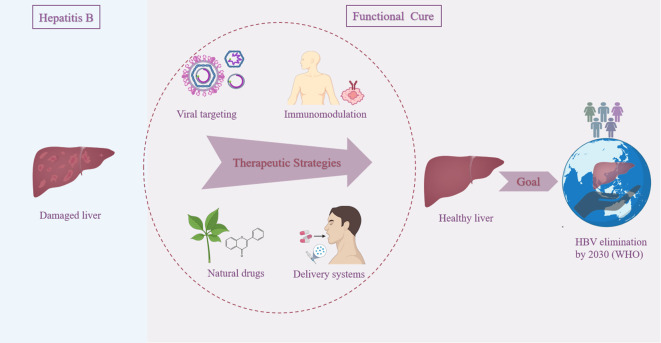

## Highlights

- Comprehensive HBV targets review: viral and host pathways.
- HBV cure barriers: cccDNA persistence and immune dysfunction
- Natural products target HBV via multi-pathway mechanisms.
- Combination therapies show promise for functional cure
- Nanodelivery advances for liver-targeted drug delivery.

## 1 Introduction

HBV is a small hepatotropic DNA virus that has infected humans for millennia (Kocher et al., 2021). Currently, approximately 300 million people worldwide are living with HBV infection, resulting in nearly one million annual deaths and making it one of the top ten global causes of mortality (Stanaway et al., 2016). In response to this substantial public health burden, the World Health Organization (WHO) established the goal of eliminating HBV by 2030, defined as a 65% reduction in mortality and 90% reduction in incidence compared to 2015 baseline levels (Cox et al., 2020). However, only 12% of countries are currently on track to achieve these WHO elimination targets (Neumann-Haefelin and Thimme, 2022). Developing an HBV cure constitutes just one essential step toward meeting the 2030 goals, necessitating immediate extensive preparations for successful implementation. Following host entry, HBV can cause either acute or chronic hepatitis B (CHB), with CHB demonstrating particularly high prevalence. Without proper clinical management, CHB frequently progresses through insidious stages of liver fibrosis and cirrhosis, ultimately leading to significantly elevated hepatocellular carcinoma risk. Despite considerable progress in HB prevention and therapeutic development in recent years, significant treatment challenges persist.

**Figure 1:**
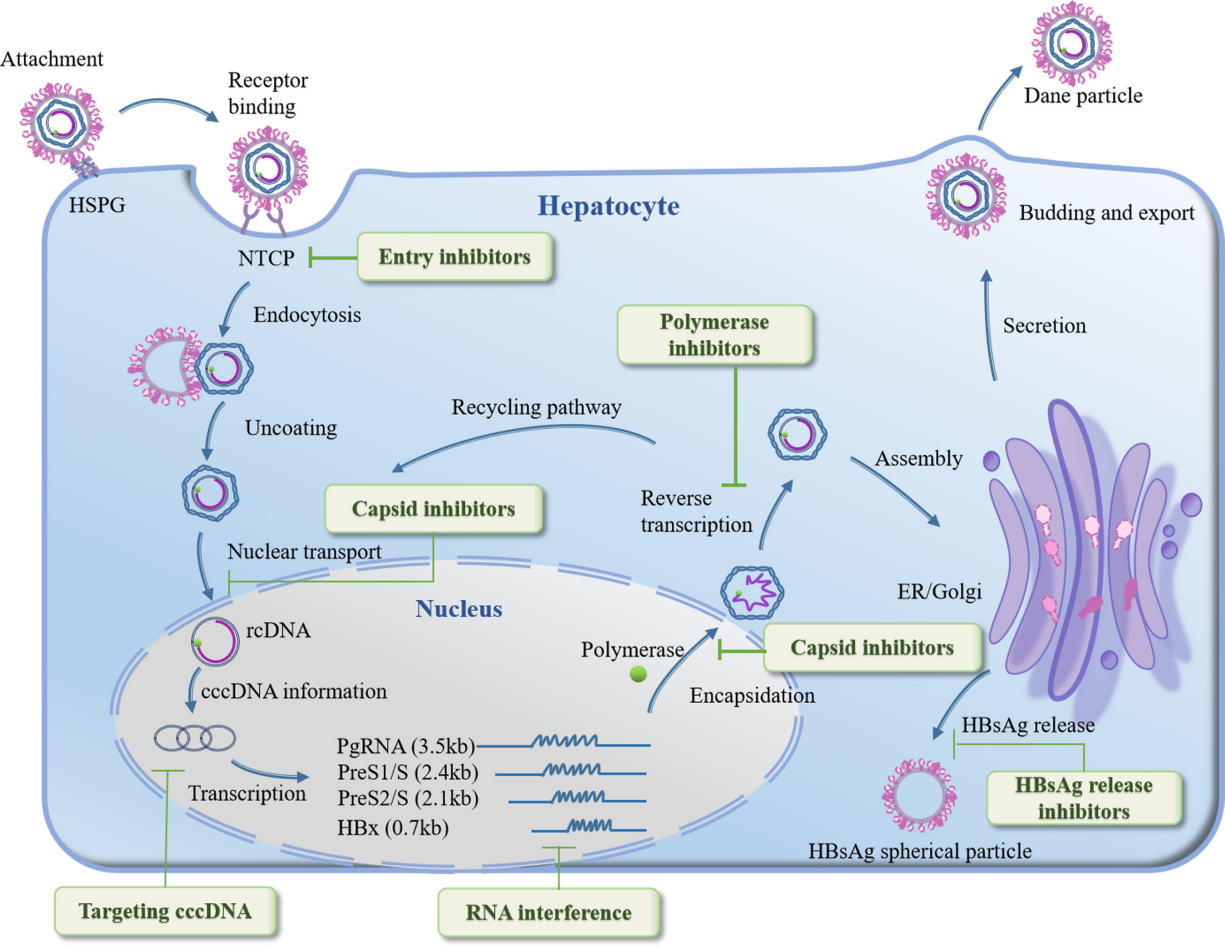
HBV life cycle and key drug targets under development.

HBV infections are extremely challenging to cure due to the virus’s high genetic diversity, persistence of the covalently closed circular DNA (cccDNA), mini-chromosome in human liver cells, HBV DNA integration into the cellular genome, and HBV’s negative impact on the host immune system (Pierra Rouviere et al., 2020). Currently, two types of treatment are approved for HBV infections. Oral nucleos(t)ides analogs(NAs) are the most commonly used anti-HBV treatment worldwide. NAs block the normal replication processes of HBV and reduce viral load by inhibiting HBV polymerase activity (Pierra Rouviere et al., 2020). NAs therapy can suppress viremia to clinically undetectable levels in up to 76% of Hepatitis B e antigen (HBeAg)-positive patients and up to 93% of HBeAg-negative patients after one year of treatment (Trépo et al., 2014). NAs have been approved against HBV (**Figure 1**). However, their functional cure rate remains<10% over long-term follow-up, due to insufficient efficacy, limited liver targeting, and/or low resistance barriers, making NAs treatment essentially lifelong (Terrault et al., 2016). Another treatment strategy is immunomodulatory therapy, which activates or regulates the host immune system to enhance immune cell recognition and killing of HBV-infected cells, thereby breaking immune tolerance. This approach achieves ≤20% functional cure rates (Kim, 2018), but is frequently associated with serious adverse reactions including cytopenia, exacerbation of neuropsychiatric symptoms (e.g., depression and insomnia), and production of thyroid autoantibodies (Tang et al., 2018). Advances in HBV research have continuously identified new therapeutic targets, including viral life cycle components and immune regulation pathways. Drug development based on these targets has shown promising results, with several candidates in clinical trials (e.g., capsid inhibitors (Ren et al., 2020) and replication inhibitors (Ma et al., 2022)) demonstrating good antiviral effects. Furthermore, novel drug delivery systems, particularly liver-targeted approaches, offer new treatment opportunities by enhancing drug precision, increasing target organ concentrations, improving therapeutic effects, and reducing adverse reactions.

This review provides a comprehensive analysis of the latest breakthroughs in HBV therapeutics, covering viral and host-directed targets, emerging small-molecule and biologic agents, natural products with multi-target mechanisms, and advanced delivery technologies. We evaluate preclinical and clinical evidence for these strategies, highlighting key challenges such as cccDNA persistence, immune reconstitution, and global treatment accessibility. By synthesizing these findings, we aim to guide future research toward rationally designed combination therapies capable of achieving durable HBV remission and, ultimately, functional cure.

## 2 New discoveries in HB treatment targets

**Figure 2:**
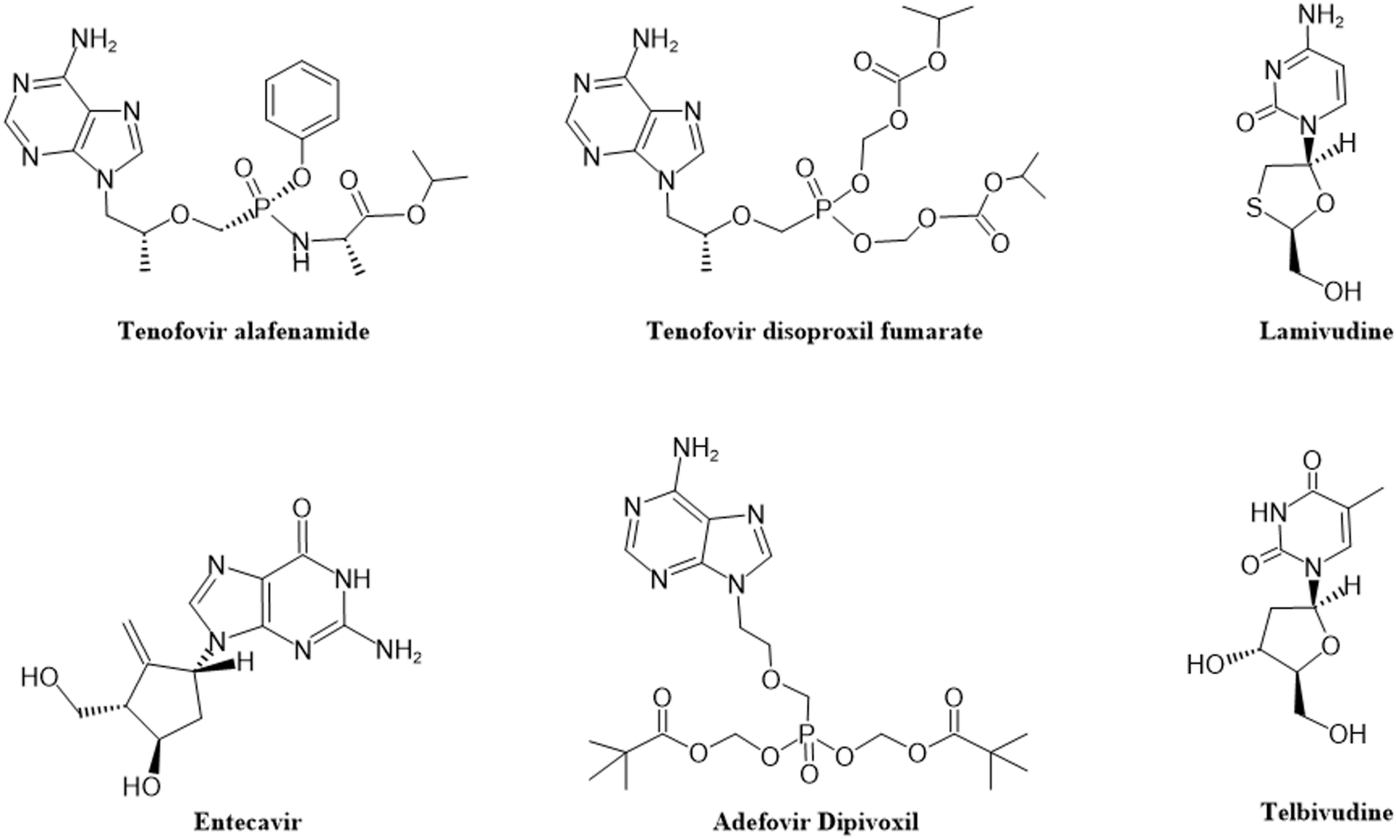
Direct-acting antiviral drugs approved for HBV treatment.

Research toward HBV cure has gained substantial momentum in recent years. Significant advances have been made in identifying HBV therapeutic targets, which are broadly classified into two categories: direct-acting antiviral targets and host-targeted therapeutic approaches. Direct-acting antiviral targets interfere with the viral life cycle by blocking viral entry, inhibiting replication, disrupting capsid assembly, or targeting cccDNA (**Figure 2**). The pursuit of these novel targets is driven by the well-recognized limitations of current standard-of-care NAs. While NAs remain the current standard of care, their inability to eliminate cccDNA and the high risk of virological relapse upon discontinuation limit their therapeutic potential (Mak et al., 2021). These novel therapeutic strategies have demonstrated clinical potential while facing persistent challenges including cccDNA persistence, immune tolerance, safety concerns, and the requirement for personalized treatment regimens. Several emerging therapeutic targets show particular promise for developing breakthrough treatment options for HBV-infected patients. This section systematically summarizes recently identified, clinically relevant therapeutic targets for HBV, highlighting those warranting further investigation to advance therapeutic development.

### 2.1 Direct-acting antiviral targets

To properly contextualize recently discovered therapeutic targets, a brief overview of the HBV lifecycle is essential. HBV infection proceeds through six key stages: (1) attachment and entry into hepatocytes, (2) conversion of relaxed circular DNA (rcDNA) to cccDNA, (3) transcription/translation of viral proteins, (4) genomic replication, (5) assembly of new virions, and (6) release of infectious particles to propagate infection (Pan et al., 2023).

#### 2.1.1 Viral entry

Viral entry inhibitors prevent HBV infection by disrupting virus-host cell receptor binding or membrane fusion processes, targeting early infection stages. The entry process could be conceptually divided into initial attachment and specific receptor engagement. Endothelial lipase acts as a bridge, connecting heparan sulfate proteoglycans and HBV, thereby facilitating the initial viral attachment. Targeting this bridge could potentially lead to the discovery of excellent entry inhibitors. Following initial attachment, a critical and specific interaction occurs between the viral preS1 domain and its high-affinity cellular receptor. Sodium taurocholate cotransporting polypeptide (NTCP) has been identified as the cellular receptor for HBV. NTCP, with its interaction domains for all these cofactors, remains the most promising drug target for the discovery of novel HBV entry inhibitors. Extensive research has identified specific binding sites within the NTCP protein for certain interaction partners, including epidermal growth factor receptor (EGFR) and NTCP oligomerization domains. However, the precise protein-protein interaction domains remain largely uncharacterized for other key partners such as interferon-induced transmembrane protein 3 (IFITM3), E-cadherin, and kinesin family member 4 (KIF4) (Zakrzewicz and Geyer, 2023). Future studies are expected to identify additional interaction partner binding sites within the NTCP protein, which would provide attractive new therapeutic targets. Beyond protein-protein interactions, structural studies provide profound insights into NTCP’s function. A groundbreaking 2024 study demonstrated that multiple residues in macaque NTCP create a structural barrier against HBV infection. Key findings include: (1) Asn86 in macaque NTCP (versus Lys86 in human NTCP) exhibits reduced ability to stabilize preS1 dynamics, and (2) long-chain conjugated bile acids in the NTCP tunnel induce steric hindrance with preS1 via their tailed chains. These structural insights establish a valuable comparative model for elucidating viral attachment restriction mechanisms between macaque and human NTCP (Shionoya et al., 2024). Collectively, these studies underscore NTCP as the cornerstone for developing novel viral entry inhibitors and provide a structural roadmap for rational drug design.

#### 2.1.2 cccDNA regulation and RNA transcription

cccDNA functions as the transcriptional template for HBV RNA synthesis, playing an essential role in viral replication. The persistence of cccDNA is a multi-faceted challenge, encompassing its initial formation, ongoing transcription, and ultimate stability within the nucleus. Luo et al. demonstrated that HBV exploits the ataxia telangiectasia and Rad3-related (ATR)–checkpoint kinase 1 (CHK1) pathway to promote cccDNA formation (Luo et al., 2020), a critical process for establishing and maintaining HBV infection. Emerging evidence suggests that the host DNA repair protein poly(ADP-ribose) polymerase 1 (PARP1) is essential for *de novo* cccDNA formation during HBV infection (Chen et al., 2022). In contrast to targeting formation, another strategy focuses on degrading cccDNA or suppressing its production. Another significant advance involves Ribonuclease H (RNase H), which effectively suppresses both HBV replication and cccDNA formation (Chauhan et al., 2021). Beyond targeting the physical DNA molecule, a prominent strategy involves modulating the transcriptional activity of the cccDNA minichromosome. These findings collectively highlight transcriptional/translational regulation of cccDNA as a promising therapeutic avenue. For example, Teng et al. identified Spliceosome-associated factor 1 (SART1) as a potent inhibitor of cccDNA transcription through its suppression of the essential transcription factor hepatocyte nuclear factor (HNF)-4α (Teng et al., 2021). Similarly, Nucleoporin 153 (NUP153) was identified as a novel host factor that enhances HBV replication by promoting cccDNA transcription via HNF4α upregulation (Jiang et al., 2025). Directly targeting the viral RNA transcripts themselves represents another powerful antiviral strategy, which can suppress viral protein production through transcript degradation or transcriptional inhibition. A key regulator bridging cccDNA transcription and downstream RNA production is the HBV X protein (HBx). The HBx, a well-characterized regulator of HBV replication, interacts with multiple transcription factors and activates signaling pathways to enhance viral replication (Slagle and Bouchard, 2016). Liu et al. identified mitochondrial glycerol-3-phosphate dehydrogenase (GPD2) as a host restriction factor that suppresses HBV replication by promoting tripartite motif-containing protein 28 (TRIM28)-mediated ubiquitination and degradation of HBx (Liu C, et al., 2023), revealing the HBx-GPD2 interaction as a potential novel target for antiviral development.

Returning to the root of persistence, the most definitive therapeutic approaches aim to directly target the cccDNA template itself. Moving beyond conventional pharmacological inhibition, recent advances in sequence-specific nuclease technologies have opened revolutionary therapeutic possibilities for directly targeting and cleaving cccDNA. Recent advances in sequence-specific nuclease technologies have opened new therapeutic possibilities against HBV. Notably, zinc finger nucleases (ZFNs), transcription activator-like effector nucleases (TALENs), and clustered regularly interspaced short palindromic (CRISPR) and CRISPR-associated protein 9 (CRISPR/Cas9) systems have demonstrated significant potential in cellular and animal models, effectively reducing both viral DNA and cccDNA levels through precise gene editing (Cai et al., 2023). Among these, the CRISPR/Cas9 system stands out due to its unparalleled programmability, high specificity, and potential for a one-time, curative therapy by permanently inactivating the cccDNA reservoir. However, the translational path from bench to bedside is fraught with substantial challenges. The foremost hurdles include ensuring the efficient and safe delivery of editing machinery to the majority of hepatocytes *in vivo*, minimizing off-target effects that could lead to genotoxicity, and addressing potential immunogenic responses to the bacterial-derived Cas9 protein. Resolving these delivery, safety, and efficacy concerns is paramount before this promising modality can be widely applied in the clinical management of chronic HBV. In summary, the multifaceted strategies targeting cccDNA—from inhibiting its formation and transcription to employing gene editing for its eradication—hold the key to achieving a functional cure for CHB.

#### 2.1.3 Viral assembly

Viral assembly represents a critical stage in HBV replication, coordinating the packaging of viral genomic material with structural proteins to generate infectious virions. The HBV core protein (HBc) serves as the fundamental building block of the HBV capsid and has emerged as an attractive target for novel therapeutics due to its multifunctional roles in the viral lifecycle. HBc appears to regulate viral processes through interactions with species-specific (but not hepatocyte-specific) host factors, which may govern HBV tropism. Of particular significance, recent studies demonstrate that HBc-directed antivirals and importin β can act synergistically to destabilize HBV capsids. This cooperative mechanism—involving host-protein interactions-not only disrupts viral assembly but may also trigger innate immune responses, revealing an unanticipated secondary mode of action for core protein allosteric modulators (CpAMs) (Kim et al., 2022).

### 2.2 Host targets

#### 2.2.1 Immunomodulatory targets

After HBV infection, the functional suppression of the host immune system is a key factor leading to chronicity. Therefore, restoring and enhancing immune responses have become crucial strategies for treatment. A central aspect of innate immune defense involves the signaling pathways that launch an antiviral state. It has been reported that mitochondrial antiviral signaling protein (MAVS) played an important role in combating HBV infection, while interferon-α (IFN-α) downregulateed MAVS expression through adenosine deaminase acting on RNA (ADAR1)-mediated RNA editing, thereby affecting antiviral efficacy (Li et al., 2021). The complex regulation of innate signaling is further reflected in the variable patient responses to exogenous IFN-α therapy, which have been linked to host genetics. Specifically, the G allele at rs2278420 and rs6509607 correlates with elevated zinc finger protein 350 (ZNF350) expression and improved pegylated IFN-α (Peg-IFN-α) response rates in HBeAg-positive chronic HBV patients, potentially through janus kinase-signal transducer and activator of transcription (JAK-STAT) signaling modulation (Guo et al., 2024). Ultimately, the efficacy of both endogenous and therapeutic interferon hinges on the concerted action of downstream interferon-stimulated genes (ISGs). Recent investigations have increasingly focused on ISGs as key mediators of antiviral activity. For instance, interferon-induced protein with tetratricopeptide repeats 3 (IFIT3) enhances IFN-α’s antiviral effects through a STAT2-dependent mechanism (Xu S, et al., 2022). Other ISGs exert their effects through more direct mechanisms: ISG20 suppresses HBV replication through direct binding to the viral Enhancer II/Core promoter (EnhII/Cp) region (Park et al., 2020), while GTPASE, an ISG, interacts directly with HBV surface antigens (HBs) proteins, reducing their intracellular levels and consequently impairing virion assembly (Quan et al., 2024).

Building upon the foundation of interferon signaling, other innate immune pathways offer distinct therapeutic opportunities. Toll-like receptors (TLRs) enhance antiviral immunity primarily through innate immune activation, positioning them as key targets for immune modulation. Complementing these findings, Ayithan et al. reported that TLR8 agonism enhances HBV-specific B cell responses in chronic HBV patients by augmenting monocyte-mediated follicular helper T cell (TFH) function, suggesting a potential role in achieving functional cure (Ayithan et al., 2021). Beyond receptor-mediated activation, the balance of inflammatory and anti-inflammatory cytokines plays a critical role in HBV pathogenesis. Liu et al. provided additional evidence that acute HBV infection downregulated the anti-inflammatory cytokine IL-37, and exogenous IL-37 alleviated liver inflammation by suppressing CD8^+^ T cell cytotoxicity, highlighting IL-37 as a potential target for immune-modulating therapy in HB (Liu Q, et al., 2023). Another layer of immune regulation involves factors that directly interface with viral components while modulating immune signaling. Monocyte chemotactic protein-1-induced protein 1 (MCPIP1) reduces HBV RNA levels, primarily through viral RNA cleavage, while also mediating IL-1β-dependent antiviral effects (Li et al., 2020). Shifting the focus to adaptive immunity, costimulatory signals are crucial for an effective T cell response. Moreover, the activation of costimulatory molecules OX40 suppresses HBV replication in a CD8^+^ T cell-dependent manner (Zhan et al., 2025). Of note, peptidoglycan-recognition protein 2 (PGLYRP2), a key innate immune sensor, acts as an age-dependent HBV eliminator by targeting viral cccDNA and nucleocapsid (Li Y, et al., 2025). In conclusion, targeting the broad spectrum of immune pathways—from innate sensors and cytokines to adaptive co-stimulation—represents a powerful and versatile approach to overcome HBV persistence and achieve immune control.

#### 2.2.2 Metabolic-regulation targets

Mounting evidence indicates that HBV exploits host metabolic pathways to facilitate its replication, making these pathways attractive therapeutic targets. The nuclear farnesoid x receptor (FXR) regulates bile acid homeostasis and plays a critical role in HBV DNA transcription. Erken et al. demonstrated that FXR-targeted agonists reduced HBsAg levels alone and further decreased HBcrAg and pregenomic RNA (pgRNA) when combined with IFN-α, with promising tolerability and safety (Erken et al., 2021). Beyond bile acid metabolism, cholesterol homeostasis is similarly subverted by the virus. In 2025, Yang’s group reported that the sterol regulatory element-binding protein (SREBP2), a key regulator of cholesterol metabolism, directly interacts with HBx protein, inhibiting its nuclear translocation and promoting extracellular secretion, thereby reducing nuclear HBx accumulation and suppressing HBV replication, which revealed a novel regulatory link between cholesterol metabolism and HBV replication (Yang F, et al., 2025). Furthermore, lipid signaling pathways are implicated in HBV-associated pathology. Moreover, it was shown that HBV infection upregulated the expression of sphingosine kinase 1 (SphK1) via the transcription factor upstream stimulatory factor 1 (USF1), increasing the production of sphingosine-1-phosphate (S1P), highlighting the SphK1-S1P axis as a potential biomarker and therapeutic target for HBV-related liver injury (Zhang et al., 2024). Together, these findings position key metabolic regulators in bile acid, cholesterol, and sphingolipid metabolism as promising host-derived targets for curbing HBV replication and mitigating virus-induced liver damage.

#### 2.2.3 Epigenetic regulatory targets

Recent advances in HBV therapy have highlighted the potential of epigenetic regulatory targets as a promising strategy. As reported by Moon et al., N6-methyladenosine (m6A) RNA methylation exerted a complex regulatory role in both HBV and host RNA, influencing viral replication, stability, and translation, while also contributing to chronic infection and liver disease progression, highlighting the critical role of m6A modification in HBV infection (Moon et al., 2024). In addition, Yang et al. suggested that the host protein TRIM28 regulated the nuclear factor kappa-light-chain-enhancer of activated B cells (NF-κB) signaling pathway, inflammatory responses, and viral replication by mediating the sumoylation of tumor necrosis factor receptor-associated factor 6 (TRAF6), indicating that sumoylation, as an important post-translational modification of proteins, serves as a key regulatory mechanism (Yang Y, et al., 2025).

#### 2.2.4 Host factor target

Targeting endoplasmic reticulum (ER) stress represents a novel strategy for anti-HBV drug development. Golzar Hossain et al. demonstrated that ER stress inhibitors significantly reduce HBc and HBsAg expression, leading to suppressed viral replication and particle production (Hossain and Ueda, 2024). Furthermore, Liang et al. provided additional evidence that HBsAg impaired autophagic flux and promoted hepatocyte proliferation by inducing ER stress and suppressing the expression of lysosome-associated membrane protein 2 (LAMP2), suggesting that restoring LAMP2 function may have antiviral effects (Liang et al., 2024). Closely linked to the processes of ER stress and autophagy, the modulation of antioxidant and autophagy regulatory pathways provides another novel strategy for HBV treatment. Excitingly, research indicated that overexpression of augmenter of liver regeneration (ALR) or the use of reactive oxygen species (ROS) scavengers could inhibit autophagy, reduce HBV protein expression, and decrease viral load (Mishra et al., 2025). In contrast to the broad inhibition of autophagy, certain host factors can co-opt the autophagic machinery to selectively target viral components for degradation. In addition, Miyakawa et al. suggested that galectin-9 (GAL9), as a product of type I ISGs, synergized with the antiviral protein viperin and the E3 ubiquitin ligase RNF13 to promote the ubiquitination of the HBc. This process recruited the autophagy receptor p62, leading to the degradation of HBc via selective autophagy and thereby inhibiting HBV replication (Miyakawa et al., 2022). Beyond protein-level degradation, host cells also employ post-transcriptional regulatory mechanisms to combat HBV. Moreover, Muchtar et al. demonstrated that the host-derived microRNA-3145 (miR-3145) is upregulated in HBV-infected primary hepatocytes and can inhibit the expression of HBsAg and HBx proteins by targeting the HBV polymerase region, thereby suppressing viral replication (Muchtar et al., 2025). Finally, enhancing the function of the endo/lysosomal compartment itself has emerged as a direct antiviral approach. The study by Yu et al. provided valuable insights into the antiviral therapeutic potential of endo/lysosomal, showing that manganese ions (Mn²^+^) and tomatidine inhibited HBV infection by activating the lysosomal activity and mechanistic target of rapamycin complex 1 (mTORC1) signaling pathway in host cells (Yu L, et al., 2025). Collectively, these diverse approaches—modulating ER stress, fine-tuning autophagy, employing microRNAs, and activating lysosomal degradation—greatly expand the arsenal of host-targeting strategies against HBV.

## 3 Future therapy for HBV

Groundbreaking progress has been made in the development of novel drugs for HB. Based on their mechanisms of action, synthetic drugs can be classified into two main strategies: those targeting the viral life cycle and host factors. The former demonstrates significant therapeutic potential by directly inhibiting key stages of viral replication and transcription, while the latter offers new possibilities for achieving a functional cure by modulating host immune responses or metabolic pathways. Meanwhile, plant-derived medicines, owing to their natural origins and multi-target mechanisms, have increasingly become a focus of HB research, particularly due to their unique advantages in anti-inflammatory and immunomodulatory effects. Below, the latest advancements in drug development, including preclinical studies and ongoing clinical trials (**Table 1**), will be discussed in detail.

**Table 1:** Direct-acting or indirect antiviral agents currently under development.

Type	Agent in development	Clinical status	NCT number	Note
Antiviral approaches				
Novel nucleoside analogs	ATI-2173	Phase I trial	NCT04248426	HBV polymerase inhibitor
	NCO-48	Phase I trial	NCT04629976	A novel TFV prodrug
	PA1010	Phase II trial	NCT05019040	HBV polymerase inhibitor
Capsid assembly modulator	JNJ-56136379	Phase II trial	NCT03361956	Bifunctional capsid/cccDNA inhibitor
	ABI-4334	Phase I trial	NCT05569941	HBc inhibitor
	ABI-H3733	Phase I trial	NCT04271592	HBc inhibitor
	ALG-000184	Phase II trial	NCT06963710	HBc inhibitor
	TQA3605	Phase II trial	NCT06644417	HBc inhibitor
	GST-HG141	Phase II trial	NCT05637541	HBc inhibitor
	EDP-514	Phase I trial	NCT04470388	HBc inhibitor
	DA-2803	Phase IV trial	NCT05957380	HBV capsid inhibitor
	ZM-H1505R	Phase II trial	NCT05484466	a novel pyrazole-class HBV core inhibitor
	XT1061	Phase I trial	NCT06280534	HBc inhibitor
	LW231	Phase I trial	NCT06311734	Bifunctional capsid assembly modulator/cGAS-STING agonist
Post-transcriptional inhibitors	BRII-835 (VIR-2218)	Phase II trial	NCT06650852	SiRNA therapy
	Imdusiran (AB-729)	Phase II trial	NCT06154278	SiRNA therapy
	JNJ-73763989	Phase II trial	NCT05275023	SiRNA therapy
	HT-101	Phase I trial	NCT06746311	SiRNA therapy
	TQA3038	Phase II trial	NCT06452693	SiRNA therapy
	HRS-5635	Phase II trial	NCT06425341	SiRNA therapy
	Bepirovirsen	Phase III trial	NCT05630807	ASO therapy
	AHB-137	Phase II trial	NCT06115993	ASO therapy
RNA stability regulation	GSK3965193	Phase I/II trial	NCT05330455	Non-canonical poly(A) polymerase inhibitor
HBsAg inhibitors	LP-128	Phase I trial	NCT05130567	-
	GST-HG131	Phase II trial	NCT06263959	-
Immunomodulatoryapproaches				
Immune stimulator	FP-02.2	Phase II trial	NCT04684914	Therapeutic vaccine
	VTP-300	Phase II trial	NCT05343481	Therapeutic vaccine
	TherVacB	Phase II trial	NCT06513286	Therapeutic vaccine
	ISA104	Phase II trial	NCT05841095	Therapeutic vaccine, synthetic long peptide (SLP^®^) technology
	CVI-HBV-002	Phase II trial	NCT04289987	Therapeutic vaccine
	JNJ-64300535	Phase I trial	NCT03463369	Therapeutic vaccine
	GS-2829	Phase I trial	NCT05770895	Therapeutic vaccine; reliable, polyclonal, genotypic cross-reactive CD8+ T cell responses
	GS-6779	Phase I trial	NCT05770895	
	ChAdOx1-HBV	Phase I trial	NCT04297917	Therapeutic vaccine
Monoclonal antibody	HepB mAb19	Phase I trial	NCT05856890	Targeting the HBV Sprotein
	GC1102	Phase III trial	NCT03519113	Reduced HBsAg levels
	HH-006	Phase I trial	NCT05275465	HBV pre-S1 inhibitor
	VIR-3434	Phase II trial	NCT06216470	a fully human monoclonal antibody
	Cetrelimab(JNJ 63723283)	Phase I trial	NCT05242445	A humanized IgG4κ monoclonal antibody targeting PD-1
	HH-003	Phase II trial	NCT05839639	A human monoclonal antibody targeting the preS1 domain
	ASC22	Phase II trial	NCT05129189	PD-L1 monoclonal antibody
PD-L1 inhibitors	AB-101	Phase I trial	NCT05960240	-
Farnesoid X receptor agonist	EYP001a	Phase II trial	NCT04365933	Non-bile acid, selective second-generation FXRagonist
	ASC42	Phase II trial	NCT05107778	Selective nonsteroidal FXR agonist
A novel agonist of innate immunity	SB 9200	Phase II trial	NCT02751996	Retinoic acid inducible gene agonist
Toll-like receptor agonist	selgantolimod	Phase II trial	NCT04891770	TLR8 agonist
	HRS9950	Phase II trial	NCT05905458	TLR8 agonist
	CB06-036	Phase I trial	NCT05828745	TLR8 agonist
	TQA3810	Phase II trial	NCT06566248	TLR8 agonist
	TQA3334	Phase II trial	NCT06706310	TLR7 agonist
Ropeginterferon alfa-2b	P1101	Phase I trial	NCT04638439	-
Inhibitor of apoptosis proteins antagonist	APG-1387	Phase II trial	NCT04568265	Enhance T cell immunity and promote apoptosis of HBV antigen-expressinghepatocytes
DirectcccDNAapproaches				
Epigenetic modulators	EPI-003	Phase I trial	NCT06745973	cccDNA inhibitor
Epigenetic silencer	Tune-401	Phase I trial	NCT06671093	Utilize lipid nanoparticles to deliver active, HBV-targeting RNA directly to hepatocytes
Gene therapy	PBGENE-HBV	Phase I trial	NCT06680232	Eliminate cccDNA and inactivate integrated HBV DNA
Others				
Novel quinone derivatives	Antroquinonol	Phase II trial	NCT03625102	Natural small molecules from Taiwan’s endemic precious species Antrodia cinnamomea

### 3.1 Synthetic drugs

#### 3.1.1 Interference with viral life cycle

##### 3.1.1.1 Entry inhibitors

Recent advances in HBV entry inhibition have identified promising therapeutic candidates. Current investigational compounds target the NTCP receptor through two distinct mechanisms: (1) direct blockade of NTCP function, or (2) disruption of NTCP-viral protein interactions. Furthermore, Yu et al. identified a novel host factor, neuropilin-1 (NRP1), which modulates HBV entry by interacting with HBV preS1 and NTCP, and demonstrated that antagonists targeting NRP1 could inhibit HBV infection both *in vitro* and *in vivo* (Yu H, et al., 2025), highlighting it as a promising new target for therapeutic development. As reported by Tanaka et al., a synthetic thiazolidinedione (TZD) derivative exhibited significant anti-HBV activity (IC_50_ = 0.3 μM, selectivity index=85) by inhibiting HBV internalization post-viral attachment, demonstrating its potential as a novel HBV entry inhibitor (Tanaka et al., 2021). It had been reported by Oshima’s group in 2023 that the semi-synthetic oxysterol Oxy185 blocked the internalization of HBV by inhibiting the oligomerization of NTCP and demonstrated favorable liver accumulation properties in a mouse model (Oshima et al., 2023). Moreover, mechanistic studies revealed that ergosterol peroxide inhibited the early fusion/endocytosis steps of hepatitis D virus (HDV) infection by blocking the binding of hNTCP to HDV, demonstrating significant anti-HDV activity *in vitro* and *in vivo*, and highlighting its therapeutic potential as an HDV entry inhibitor (Chiou et al., 2024). We believe this provided a promising insight for the treatment of HBV infection. In addition, Takemori et al. suggested that a monoclonal antibody, N6HB426-20, effectively blocked HBV entry into host cells by targeting the extracellular domain of NTCP, and provided long-term prevention of HBV viremia in a mouse model (Takemori et al., 2022). Interestingly, a study found that the mechanistic target of rapamycin (mTOR) inhibitor everolimus blocked HBV/HDV entry by directly inhibiting the function of NTCP, offering a potential strategy for developing novel drugs targeting viral entry (Saran et al., 2023). Collectively, these entry inhibitors offer a promising strategy to prevent initial infection. However, their clinical utility is likely confined to combination therapies or prophylaxis, as their efficacy against established chronic HBV is expected to be minimal.

##### 3.1.1.2 Directly targeting cccDNA

Targeting cccDNA has emerged as a central strategy in HB therapeutic research, with the ultimate goal of achieving a functional cure through either transcriptional silencing or direct degradation. Although substantial challenges persist, cccDNA-directed therapies represent the most promising avenue for complete HBV eradication. Current efforts are exploring diverse chemical space and repurposing existing drugs to identify effective cccDNA-targeting agents. As reported by Wang et al., a novel small-molecule cccDNA inhibitor (ccc_R08) that specifically reduced HBV cccDNA levels and significantly decreased HBV DNA and antigen levels both *in vitro* and in mouse models (Wang et al., 2023). In contrast to cccDNA-targeting approaches, cyclocytidine hydrochloride significantly reduced the synthesis of rcDNA by inhibiting the activity of HBV DNA polymerase, offering a potential rationale for the “new” application of this “old” drug in anti-HBV therapy (Wang X, et al., 2022). Moreover, by developing a highly sensitive cccDNA screening system, Ren et al. discovered that the antihistamine bilastine and its structural analogs (such as pitolisant and nizatidine) could directly suppress cccDNA levels (Ren E. C, et al., 2023). These drugs, despite lacking obvious structural similarities, all regulate HBV cccDNA by targeting different stages of its lifecycle, such as maintenance, replication, or transcription, through distinct mechanisms like direct degradation, polymerase inhibition, or host factor interference. This diversity highlights multiple strategies for cccDNA-targeted drug development, and combining drugs with different mechanisms may further enhance this advantage. In summary, directly targeting cccDNA holds the unique advantage of attacking the viral reservoir’s root cause. However, this approach is still in its infancy, with most candidates in early preclinical stages and their precise mechanisms of action and delivery to the nucleus remaining significant hurdles.

##### 3.1.1.3 Targeting viral transcripts

The development of RNA interference (RNAi) technology in the treatment of HB has progressed rapidly. Currently, small interfering RNA (siRNA) therapies have entered clinical trial stages, demonstrating favorable safety and antiviral efficacy. It had been reported by Mak’s group in 2025 that the short-term siRNA therapy (such as ARC-520 and JNJ-3989) significantly suppressed HBsAg expression in patients with CHB and produced long-term effects lasting up to 6 years in some patients. Additionally, the annual decline rate of HBsAg levels was faster, and age was negatively correlated with the reduction in HBsAg (Mak et al., 2025). A recent study showed that HT-101, as an siRNA drug, could efficiently inhibit HBV transcription, significantly reduce HBsAg, HBeAg and HBV DNA levels, and induce the production of anti-HBs (Zhang et al., 2025). Beyond siRNA, alternative modalities are emerging. It is noteworthy that SAG-524, a novel oral HBV RNA destabilizer targeting poly(A)-specific ribonuclease domain-containing protein 5 (PAPD5), significantly reduced HBV RNA and HBsAg levels with favorable safety and combination potential in preclinical studies (Tanaka, 2025). Complementing these direct transcript-targeting approaches, drug repurposing provides a faster, lower-cost strategy for developing new HBV treatments. For instance, the anticancer agent cabozantinib impeded HBV transcription and replication by disrupting STAT-3-cccDNA interaction via hepatocyte growth factor (HGF)-MET-STAT3 axis inhibition (Funato et al., 2023). Furthermore, Pimobendan, a cardiac drug, suppressed HBV promoter activity and reduced cccDNA transcription, providing potential clues for developing novel anti-HBV therapeutics (Yuan et al., 2022). In summary, transcript-targeting therapies, especially siRNAs, excel at antigen suppression but face key limitations: they cannot eradicate cccDNA, typically require injections, and their effects may not be durable. Oral alternatives offer convenience but often with reduced potency.

##### 3.1.1.4 Targeting viral replication

Research on RNase H inhibitors remains at an early stage, though several lead compounds have demonstrated antiviral activity. Li et al. discovered a series of amide-containing α-hydroxytestosterone compounds, which exhibited potent and specific inhibitory effects against RNase H. Future research should focus on addressing the cytotoxicity of these compounds (Li et al., 2020). In contrast, more promising drug-like properties have been reported for other chemotypes. Several studies have indicated that a series of N-hydroxypyridinedione (N-HPD) compounds show effective RNase H inhibitory activity (Moianos et al., 2024; Giannakopoulou et al., 2024). Similarly, Woodson et al. observed that the novel N-HPD compounds they developed exhibited excellent anti-HBV activity and selectivity indexes of 526 and 1,071, along with favorable pharmacological properties, including high solubility, low cytotoxicity, and good stability in human liver microsomes, making them suitable for further *in vivo* studies (Woodson et al., 2024).

Parallel to the pursuit of novel viral targets, the optimization of established mechanisms continues. Novel NAs have shown enhanced antiviral activity, lower resistance rates, and improved safety profiles in the development of drugs. Currently, extensive studies are focused on exploring and developing next-generation drugs. Onitsuka et al. proposed that a series of 4′-Azido-thymidine/4′-Azido-2′- deoxy-5-methylcytidine derivatives had significant anti-HBV activity (EC50: 0.63 and 5.99 μm) with no remarkable cytotoxicity (Onitsuka et al., 2020). In addition, Zhang et al. designed and synthesized an o-methylbenzyl group (1a). In HBV-infected ducks, it showed potent anti-HBV activity, good tolerability and safety, and higher liver exposure of the active metabolite than tenofovir alafenamide fumarate (TAF) (Zhang Q, et al., 2022). Excitingly, a research group recently developed E-CFCP, a novel long-acting anti-HBV drug that inhibited viral replication with low nephrotoxicity and negligible impact on hepatocyte senescence (Ishibane et al., 2022; Takamatsu et al., 2023). Further X-ray crystallography studies showed interactions between E-CFCP-triphosphate and the dNTP-binding site of HBV reverse transcriptase, which should help further design antiviral NAs against HBV (Yasutake et al., 2024).

##### 3.1.1.5 Capsid assembly inhibitors

Small-molecule capsid assembly modulators (CAMs) can broadly be classified into heteroaryldihydropyrimidines and sulfamoylbenzamides, which have become a research hotspot in anti-HBV therapy by targeting the HBc to disrupt viral capsid assembly. Lee et al. synthesized novel sulfamoylbenzamides as CAMs of HBV and found that compounds 3 and 8 showed potential therapeutic applicability against HBV based on their effects and safety profiles (Lee et al., 2021). Similarly, Na et al. reported a novel series of HBV CAMs based on NVR 3-778, a potent CAM belonging to the SBA class. And the lead compound (KR-26556) exhibited improved pharmacological activity and was examined through molecular docking studies (Na et al., 2020). Due to the poor solubility of NVR 3–778 and other obstacles hindering its development, Wang et al. designed a series of novel derivatives through structural optimization. Among them, compound 7b exhibited anti-HBV activity comparable to NVR 3–778 while significantly improving water solubility, providing a foundation for further structural optimization (Wang S, et al., 2022). However, the study by Yuen et al. was limited by hepatotoxicity. In detail, a small-molecule capsid inhibitor, AB-506, was observed to cause severe transaminase elevation in severe transaminase elevation in Asian participants during Phase I clinical trials, leading to the termination of its development program (Yuen et al., 2022).

Heteroaryldihydropyrimidines target the allosteric site of HBc through their dihydropyrimidinone core structure and aromatic heterocyclic modifications, effectively inhibiting capsid assembly and reducing viral replication, with representative drugs including JNJ-64530440 (Gane et al., 2022). In addition, through structural optimization, Zhao et al. designed a series of heteroaryldihydropyrimidines derivatives, among which compound 6a-25 showed potent anti-HBV activity (EC_50_, half maximal effective concentration=0.020μm) and significantly improved metabolic stability (half-life=108.2min) (Zhao et al., 2023). Moreover, linvencorvir, an HBc allosteric modulator based on a heteroaryldihydropyrimidine core structure, exhibited potent anti-HBV activity and high metabolic stability. However, it failed to achieve functional cure as monotherapy and requires combination with other agents (e.g., siRNA or immunomodulators) to further enhance therapeutic efficacy (Hou et al., 2023; Zhang W, et al., 2023).

Beyond these two main classes, a diverse array of novel CAM scaffolds is emerging. These include Freebudine, which has demonstrated significant antiviral activity and a favorable safety profile in clinical trials (Li X, et al., 2025), as well as 1,2,3,4-tetrahydroquinoxaline, benzimidazole, phthalazinone, and tetrahydropyrazolopyrazine derivatives, all designed to engage the hydrophobic pocket of HBc (Hwang et al., 2023; Du et al., 2024; Yang L, et al., 2024; Kou et al., 2023). The field is also exploring innovative strategies, such as the coreSN fusion protein which links HBc to a nuclease for capsid-specific degradation (Bendahmane et al., 2024), and the repurposing of the antifungal drug ciclopirox into novel HBc inhibitors via QSAR analysis (Mohebbi et al., 2023).

##### 3.1.1.6 HBsAg production and secretion inhibitors

Currently, the development of HBsAg secretion inhibitors has become a key focus in HBV therapeutic research globally. It is noteworthy that the Pyrazolo[3,4-d]pyrimidine-based neplanocin analogues showed potent dual anti-HBV activity by inhibiting both HBV DNA replication and HBsAg secretion, while exhibiting low cytotoxicity and a unique mechanism of action (Kasula et al., 2025). It had been reported by Shrestha’s group in 2024 that lycorine targeted the HBV small envelope protein, specifically its amphipathic α-helical region (W156 to R169), thereby inhibiting subviral particle (SVP) biosynthesis and significantly reducing HBsAg secretion (Shrestha et al., 2024). Song et al. proposed that the dihydroquinolizinone derivative demonstrated significant inhibition of HBsAg production with markedly reduced neurotoxicity and favorable pharmacokinetic properties (Song et al., 2024). These promising findings warrant further research focusing on elucidating their detail mechanisms of action, which will facilitate the rational development of more effective therapeutic compounds.

#### 3.1.2 Host-targeting agents

Current research on host-targeting therapies for HB primarily focuses on two key approaches: modulating host immunity (e.g., TLR agonists and monoclonal antibodies) and targeting HBV-associated host factors. Meanwhile, CRISPR-based gene editing strategies directed against HBV-dependent host genes are advancing from laboratory research toward clinical translation (Zhang J, et al., 2023).

##### 3.1.2.1 TLR agonists

TLR agonists represent a promising class of immunomodulators for CHB, targeting various TLRs to activate innate immune responses. As reported by Yuen et al., the TLR7 agonist RO7020531 exhibited safety and tolerability across all tested doses in both healthy volunteers and CHB patients during a phase I trial, supporting its further development as an immunotherapeutic approach for CHB infection (Yuen et al., 2023a). Similarly, Ibrahim Omer et al. observed that TLR7 agonist vesatolimod showed a favorable safety and tolerability profile in CHB patients, effectively inducing ISG expression though failing to achieve significant HBsAg reduction (Omer et al., 2023). Complementing the clinical focus on TLR7 and TLR8, preclinical research on TLR2 activation has shown significant promise. The TLR2 agonist Pam3CSK4 (a synthetic tri-palmitoylated lipopeptide) demonstrates potent anti-HBV activity, significantly suppressing both HBV RNA and cccDNA levels via the TLR1/2-NF-κB pathway. Notably, Pam3CSK4 shows synergistic effects when combined with flap endonuclease-1 (FEN-1), IFN-α, or the experimental kinase inhibitor 1C8, highlighting its potential as an immunomodulatory therapy for HB (Desmares et al., 2022). In addition, the oral TLR8 agonist selgantolimod could induce serum cytokine changes and immune cell redistribution, while also remodeling the liver immune microenvironment by downregulating NTCP expression and inhibiting HBV entry (Gane et al., 2023; Roca Suarez et al., 2024). It is noteworthy that conditioned media from peripheral blood mononuclear cells (PBMCs) stimulated by dual-acting toll-like receptor agonists (e.g., R848 and CL413) could effectively suppress HBsAg secretion (Janovec et al., 2020). This inhibitory effect is associated with the synergistic action of multiple cytokines, multispecific TLR agonists to achieve functional cure.

##### 3.1.2.2 Monoclonal antibodies

Monoclonal antibodies (MABs) have emerged as a research hotspot in hepatitis B due to their unique therapeutic advantages, which include the direct clearance of HBsAg, the versatility of bispecific designs, and advancements driven by bioengineering. Several innovative MAB-based strategies highlight this progress. For instance, a conventional anti-HBsAg MAB demonstrated significant efficacy in both preventing and treating chronic HBV infection in preclinical models, effectively blocking viral entry and reducing viral load (Burm et al., 2022). Beyond direct neutralization, MABs are also being harnessed for targeted delivery. A novel hepatocyte-specific drug delivery system (10M-D42AN) effectively suppressed HBV proliferation in mice by using an anti-asialoglycoprotein receptor antibody to target host factors essential for HBV infection (Ide et al., 2022). The functional capabilities of MABs have been further expanded through sophisticated engineering. Xie et al. developed a cell-penetrating bispecific antibody that simultaneously targets HBsAg PreS1 and HBcAg, utilizing the cell-penetrating peptide R9-TAT for intracellular delivery to inhibit HBV replication and secretion (Xie et al., 2025). Similarly, Fc domain engineering is being exploited to enhance effector functions. Jiang et al. developed a dual-domain engineered antibody, 73-DY, which significantly enhanced viral neutralization and clearance through synergistic Fab and Fc engineering, representing a new generation of high-potency antibody therapy (Jiang et al., 2024). Overall, MABs provide a potent and specific modality for HBV treatment, excelling in antigen clearance and prophylaxis. Their broader application, however, is constrained by high production costs, the risk of viral escape, and the inherent difficulty of targeting intracellular viruses with conventional antibodies.

##### 3.1.2.3 Other host-targeting agents

Research into other host-targeting agents has uncovered immunomodulatory, metabolic, and innate immune pathways as viable therapeutic avenues. It has been found that an anti-(programmed death ligand 1 (PDL1))-based IFN fusion protein targeting the liver successfully broke immune tolerance in a CHB mouse by simultaneously blocking the programmed death 1 (PD1)/PDL1 immune checkpoint and activating dendritic cells, and enhanced anti-HBsAg immune response when combined with a HB vaccine (Meng et al., 2023). Complementing this targeted immunomodulation, broader immune activation is also being explored. A phase II trial evaluating the efficacy and safety of a retinoic acid-inducible gene 1 agonist in CHB treatment, demonstrating its dose-dependent reduction of both HBV DNA and HBsAg levels with favorable tolerability (Yuen et al., 2023b). Notably, probiotics and their derived spermidine promote HBV clearance via autophagy-enhanced IFN-γ^+^CD4^+^T cell immunity, highlighting the therapeutic potential of probiotics and spermidine for the functional cure of HBV patients and offering valuable insight into the immune mechanism of microbiota-mediated HBV clearance (Wang T, et al., 2024).

Beyond immunomodulation, targeting host factors involved in viral restriction offers another strategy. APOBEC3G (A3G) is a potent host defence factor, which inhibits HIV-1 replication via its G-to-A hypermutation activity (Harris and Liddament, 2004). A study synthesized a novel N-phenylbenzamide derivative IMB-0523 that significantly suppressed both wild-type and drug-resistant HBV replication by elevating intracellular A3G levels, while demonstrating potent antiviral activity and low toxicity in animal models (Cui et al., 2020). Additionally, Shi et al. has demonstrated that modulation of selenium metabolism could suppress HBV replication and hepatotoxicity. Specifically, selenium donors (Na2SeO3) inhibited HBV replication and alleviated HBx protein-associated liver toxicity by promoting apoptosis and suppressing ferroptosis pathways (Shi et al., 2023). Notably, it was identified that the small-molecule compound iCDM-34 through computational screening, which suppressed HBV DNA replication by activating the aryl hydrocarbon receptor (AhR) and exhibited synergistic antiviral effects when combined with entecavir (ETV) (Furutani et al., 2023). In essence, targeting host factors provides a promising strategy to circumvent viral resistance. This approach nonetheless faces hurdles, including the potential for on-target/off-tissue toxicity and the complex safety profile associated with immune pathway modulation.

### 3.2 Natural products

Natural products showed great promise in HBV therapy, mainly including active ingredients such as phenylpropanoids, flavonoids, polysaccharides and terpenoids (**Table 2**). These compounds could exert anti-HBV effects through direct inhibition of viral replication, modulation of host immunity or protection of hepatocytes. In addition, a variety of Chinese herbal formulas and proprietary Chinese medicines have been applied in clinical practice, and their multi-component synergistic mechanism of action has received widespread attention. Although some of the mechanisms still need to be explored in depth, natural products and their derivatives provided important resources for the development of novel anti-HBV drugs, and standardization studies and clinical validation need to be strengthened in the future.

**Table 2:** Natural product components for HBV: structures, classifications, sources, and mechanisms of action.

Active compound	Group	Plants/Sources	Mechanisms	Systems	Limitations	References
Sphondin	Phenylpropanoids	Heracleum laciniatum	inhibit HBsAg production	HepG2-NTCP cell and Primary human hepatocytes	No effect on cccDNA levels	(Ren F, et al., 2023)
Psoralen	Phenylpropanoids	Cullen corylifolium (L.) Medik (syn. Psoralea corylifolia L)	suppress HBV RNA transcription and HBc expression.	HepG2.2.15 cells	poor water solubility; the absence of primary hepatocyte-based experiments	(Ma et al., 2022)
Furanocoumarins Fc-20 and Fc-31	Phenylpropanoids	Grapefruit	Inhibit cccDNA and HBsAg production	HepG2.2.15 and HepAD38 cells and PHH	Lack of *in vivo* and pharmacokinetic studies	(Tyagi et al., 2024)
Imperatorin	Phenylpropanoids	Angelica dahurica	Inhibit cccDNA and HBsAg production	HepG2-NTCP cell and Primary human hepatocytes	Lack of long-term efficacy and drug resistance evaluation;limitations of experimental models.	(Ren et al., 2024)
esculetin derivatives	Phenylpropanoids	Aesculus hippocastanum	Inhibit HBeAg and HBsAg production	HepG2.2.15 cells	No investigation of HBV DNA replication, cccDNA stability, or HBV RNA transcription.	(Ye et al., 2023)
Dicoumarol	Phenylpropanoids	Melilotus officinalis	Inhibit the transcription of cccDNA	HepG2-NTCP cells	Poor safety profile; non-specific targeting.	(Cheng et al., 2021)
(+)-Lariciresinol	Phenylpropanoids	Roots of Isatis indigotica Fortune ex Lindl	inhibit HBV DNA replication	HepG2.2.15 cells	Lack of cccDNA level detection.	(Yang et al., 2022)
Resveratrol	Phenylpropanoids	Polygonum cuspidatum, grapes, peanuts, mulberries	inhibit HBV replication	HepG2 and HepG2.2.15 cells	Lack of in-depth studies on the mechanisms by which RVT interferes with HBV replication	(Pan et al., 2022)
Curcumin	Phenylpropanoids	Curcuma longa	interrupted HBV entry	HepG2-hNTCP	Limitations of the Cell Models	(Thongsri et al., 2021)
polyphenols resveratro; oleuropein	Phenylpropanoids	Grapes; Olea europaea	Improve the function of T cells	T cells	Limitations of the *In Vitro* Model	(Acerbi et al., 2021)
lithospermic acid	Phenylpropanoids	Salvia miltiorrhiza	autophagy regulation	HepG2.2.15cells	Limitations of the Cell Models	(Zhu et al., 2023)
Pterostilbene	Phenylpropanoids	blueberries and grapes	inhibit HBV DNA replication	HepG2.2.15cells	Inadequate validation of antiviral mechanism and lack of cccDNA assessment	(Wang et al., 2021)
a polysaccharide from Radix Isatidis	polysaccharide	Isatis indigotica Fortune	Reduce the level of HBsAg and the viral load of HBV DNA	HepG2.2.15cells	Lack of *In Vivo* Efficacy and Safety Data	(Wang T, et al., 2020)
a polysaccharide from Thais clavigera	Polysaccharides	Thais clavigera	Inhibit cccDNA and HBsAg production	HepG2.2.15cells	The limitations of *in vivo* experiments.	(Tang et al., 2021)
emodin	anthraquinone	Polygonum multiflorum, Rheum palmatum, and Cassia obtusifolia	inhibited HBV DNA replication and the secretion of HBsAg	HepG2 cells	Lack of *in vivo* experiments and Limitations of using a single cell line	(Wang Y, et al., 2024)
Amentoflavone	Flavonoids	Ginkgo biloba; Platycladus orientalis	interrupted HBV entry	HepG2-hNTCP-C4 cells and PXB-cells	Lack of validation in animal models	(Aoki-Utsubo et al., 2023)
swertisin	Flavonoids	Iris tectorum Maxim	Inhibit cccDNA and HBsAg production	HepG2.2.15 cells and HepG2-NTCP cells	lack of cccDNA assessment	(Xu et al., 2020)
Baicalein	Flavonoids	Scutellaria Baicalensis Georgi	Inhibit cccDNA, HBeAg and HBsAg production	HepG2 and HepG2.2.15 cells	Lack of Pharmacokinetic and Toxicity Studies	(Niu et al., 2025a)
Baicalin	Flavonoids	Scutellaria Baicalensis Georgi	inhibited HBV transcription	entecavir-resistant HBVrtM204V/rtLl80M transfected HepG2 cells	Lack of Pharmacokinetic and Toxicity Studies	(Xia et al., 2020)
baicalin	Flavonoids	Scutellaria Baicalensis Georgi	inhibit HBV replication	HepG2 and HepG2.2.15 cells	Lack of Pharmacokinetic and Safety Evaluations	(Niu et al., 2025b)
quercetin	Flavonoids	Sini Decoction Plus Ginseng Soup	Inhibit HBeAg and HBsAg production	Hep3B and HepG2.2.15 cells	Lack of Animal Models and Limitations of Network Pharmacology	(Hao et al., 2024)
Solamargine	Alkaloids	Solanum	inhibited HBV core promoter activity and reduced pregenomic RNA level	Huh7 and HepG2.2.15 cells	Lack of Animal Models andSafety Evaluations	(Chen et al., 2024)
Skimmianine	Alkaloids	Rutaceae	interrupted HBV entry	a high-throughput screening system using HiBiT-tagged recombinant HBV	Lack of Animal Models andSafety Evaluations	(Yoshita et al., 2024)
matrine	Alkaloids	Sophora flavescent	Inhibited HBV Replication and Modulated Immune Response	HepG2.2.15 cells	Limitations of *In Vitro* Studies	(Zhou et al., 2022)
Oxymatrine	Alkaloids	Sophora flavescent	Inhibited Transcription Factor Expression	HepG2.2.15 cells	Lack of *In Vivo* Data	(Wang F, et al., 2024)
PAC5	Terpenes	Phyllanthacidoid A	Inhibit cccDNA and Modulated Immune Response	HepG2.2.15 cells, HepAD38 cells and Primary human hepatocytes	Mechanism not fully elucidated	(Zuo et al., 2022)
Ciliatoside A	Terpenes	Peristrophe japonica	InhibitHBsAg expression and cccDNA transcription; Induced HBc autophagic degradation	HepG2.2.15 cells	Lack of safety studies and bioavailability research	(Fang et al., 2023)
Syringopicroside	Terpenes	Syringa oblata	Inhibit cccDNA, HBeAg and HBsAg production	HepG2.2.15 cells	Lack of long-term toxicity studies and in-depth mechanistic research	(Zhang X. W, et al., 2022)
Asiatic acid	Terpenes	Centella asiatica	Inhibit cccDNA transcription	HepG2-NTCP cells and Primary human hepatocytes	Lack of pharmacokinetic studies	(Li et al., 2024)
pentacyclic lupane-type betuli-derived triterpenoids	Terpenes	betulin	interrupted HBV entry	HepG2 cells	Lack of *in vivo* experimental validation and toxicity assessment	(Kirstgen et al., 2020)
taraxasterol	Terpenes	Taraxacum officinale F.H.Wigg	Inhibit cccDNA, HBeAg and HBsAg production	HepG2.2.15 cells	Lack of cytotoxicity assessment and validation in animal studies	(Yang et al., 2020)
betulinic acid	Terpenes	Betula pendula	inhibit cccDNA, HBeAg and HBsAg production	HepG2.2.15 cells	Lack of long-term antiviral efficacy studies	(Chen et al., 2024)
Total saponins	saponins	Abrus cantoniensis Hance	inhibit cccDNA, HBeAg and HBsAg production	HepG2.2.15 cells	lack of cccDNA assessment	(Yao et al., 2020)
Polyoxygenated Tropolones	Tropolone derivatives	Thuja plicata	Inhibit cccDNA transcription	HepDES19 and HepG2.2.15 cells	Lack of *in vivo* experimental validation and toxicity assessment	(Schiavone et al., 2022)

#### 3.2.1 Monomeric compounds

##### 3.2.1.1 Phenylpropanoids

Phenylpropanoids, a large class of phenylalanine-derived natural products including coumarins, lignans, and phenolic acids, are widely investigated for anti-HBV potential due to their antiviral, antioxidant, and anti-inflammatory activities (Neelam et al., 2020). In recent years, coumarin analogs have made breakthroughs in anti-HBV therapy, with their mechanism of action mainly focusing on key aspects such as targeting HBx protein degradation and regulating cccDNA transcription. Several studies have demonstrated distinct molecular mechanisms for different coumarin derivatives. For instance, the furanocoumarin derivatives Fc-20 and Fc-31 significantly inhibit HBV cccDNA transcription, viral replication, and antigen secretion by targeting HBx proteins and promoting their proteasomal degradation through binding to Ala3, Arg26, and Lys140 residues, while demonstrating synergistic antiviral effects when combined with entecavir (Tyagi et al., 2024). Similarly, Ren et al. proposed that eumelanocortin effectively inhibited HBV promoter activity, significantly reduced HBsAg expression, HBV DNA levels, and cccDNA transcriptional activity by directly binding to extracellular signal-regulated kinase (ERK) proteins and interfering with cAMP response element-binding protein (CREB) activation, providing a new strategy for targeting the ERK-CREB axis in the treatment of CHB (Ren et al., 2024). Moreover, psoralens were found to inhibit HBV RNA transcription and viral replication by down-regulating the expression of the transcription factor forkhead box-O 1 (FOXO1) and decreasing the binding of the FOXO1-peroxisome proliferator-activated receptor gamma coactivator 1α (PGC1α) complex to the enhancer II region of the HBV prenuclear/core promoter (Ma et al., 2022). In addition to this, sphondin could promote the proteasomal degradation of HBx, thereby reducing the recruitment of HBx to cccDNA and effectively inhibiting cccDNA transcriptional activity and HBsAg expression (Ren F, et al., 2023). In terms of structural optimization, it was found that HBeAg expression was significantly inhibited by the introduction of a morpholine moiety at the 7-position of esculetin (IC_50_, half maximal inhibitory concentration=15.8 μM), and HBsAg expression was effectively inhibited by the introduction of a 2-methylimidazole moiety (IC50 = 21.4 μM), and these structural modifications not only enhanced the anti-HBV activity, but also improved metabolic stability (Ye et al., 2023).

Beyond coumarins, other phenylpropanoids also show promise. The lignan (-)-lariciresinol from Panax quinquefolius extract significantly reduced HBV RNA levels and blocked DNA replication by modulating HNF-1α-mediated transcriptional repression (Yang et al., 2022). Moreover, it was shown that resveratrol markedly reduced the level of HBV replication in HepG2.2.15 cells by inhibiting miR-155 expression and activating cellular autophagy pathway (Pan et al., 2022). Of note, curcumin effectively inhibits HBV attachment and internalization in imHC and HepaRG cells by specifically binding to the NTCP receptor, reducing viral load, HBeAg/HBcAg expression, and HBV DNA/cccDNA levels (Thongsri et al., 2021). It had been reported by Zhu’s group in 2023 that lithospermic acid significantly enhanced autophagic flow by inhibiting the phosphoinositide 3-kinase/ak strain transforming/mechanistic target of rapamycin (PI3K/AKT/mTOR) pathway and inhibited HBV DNA replication and antigen expression in a dose-dependent manner in HepG2.2.15 cells and HBV-(hydrodynamic delivery-based infection (HDI)) mouse models, demonstrating that the anti-HBV effect was completely dependent on the autophagy activation mechanism (Zhu et al., 2023). Pterostilbene blocked HBV DNA replication by specifically targeting the ribonucleotide reductase M2 subunit (RRM2) and was effective against lamivudine-resistant strains, and its antiviral effect can be reversed by exogenous dNTP, confirming RRM2 as the core target for its dual antiviral action (Wang et al., 2021).

##### 3.2.1.2 Flavonoids

Leveraging their antioxidant, anti-inflammatory, and immunomodulatory properties, flavonoids are promising broad-spectrum antiviral agents and have demonstrated multi-target potential in HBV therapy (Dhankhar et al., 2025). Amentoflavone dose-dependently inhibited HBV infection by blocking the binding of HBV preS1 to the host cell surface receptor NTCP, thus identifying a potential scaffold for novel NTCP-targeting inhibitors (Aoki-Utsubo et al., 2023). Furthermore, baicalin inhibited the synthesis of HBV RNA and viral proteins by down-regulating the HNF4α-HNF1α transcriptional regulatory axis, thereby effectively blocking HBV replication (Xia et al., 2020). Similarly, baicalin inhibited HNF-dependent HBV transcription and replication through sequential activation of estrogen receptor α (ERα)-liver kinase B1 (LKB1)-adenosine monophosphate-activated protein kinase α (AMPKα) signaling axis (Niu et al., 2025). In addition, baicalein inhibited HBV intracellular transport and replication by targeting the coiled-coil domain-containing protein 88A (CCDC88A)-AKT-mTOR signaling pathway and activating autophagy, thereby significantly reducing HBsAg, HBeAg and HBV-DNA levels (Niu et al., 2025). Of note, it has been reported that quercetin, a key active ingredient in Sini Decoction Plus Ginseng Soup, significantly inhibited the proliferation of HBV-associated hepatocellular carcinoma cells and reduced the expression level of HBsAg/HBeAg by targeting the inhibition of the cyclin-dependent kinase 1 (CDK1)/Cyclin B1 (CCNB1) cell cycle regulatory pathway, revealing its dual anti-HBV-HCC mechanism (Hao et al., 2024).

##### 3.2.1.3 Alkaloids

Alkaloids are a class of naturally occurring, nitrogen-containing compounds known for their structural diversity and potent biological activities. Alkaloids exhibited multi-target and multi-pathway anti-HBV characteristics. Recently, it has also been reported that solamargine exerted antiviral effects by specifically binding to the transcription factor myeloid zinc finger protein 1 (MZF1), inhibiting HBV core promoter activity and reducing pgRNA levels (Chen W, et al., 2024). Moreover, it has been shown that matrine inhibited HBV replication by suppressing protein kinase C (PKC) phosphorylation and blocking the activation of the mitogen-activated protein kinase (MAPK)/activating transcription factor 2 (ATF2) signaling pathway, while also enhancing the expression of C-X-C motif chemokine ligand 8 (CXCL8) to modulate immune responses (Zhou et al., 2022). Interestingly, oxymatrine has been shown to effectively suppress HBV DNA replication, 3.5-kb RNA transcription, and HBsAg/HBeAg secretion by activating ERK1/2 phosphorylation while significantly downregulating hepatic nuclear factors HNF1α and HNF4α (Wang F, et al., 2024). Yoshita et al. developed a high-throughput screening system using HiBiT-tagged recombinant HBV and identified the compound skimmianine as a potent inhibitor of HBV infection by blocking retrograde transport of viral capsids post-internalization (Yoshita et al., 2024).

##### 3.2.1.4 Terpenes

Terpenes, also known as terpenoids, constitute a large and diverse class of organic compounds derived from five-carbon isoprene units. They are the major constituents of essential oils from many plants and exhibit a wide range of pharmacological effects (Papada, 2025). Terpenes have made significant progress in anti-HBV drug development. The sesquiterpenoid compound PAC5 showed potent anti-HBV and anti-SARS-CoV-2 activity both *in vitro* and *in vivo* by activating the heterogeneous nuclear ribonucleoprotein A2B1 to trigger the TANK-binding kinase 1 (TBK1)-IFN regulatory factor 3 (IRF3) signaling pathway, thereby promoting type I interferon production (Zuo et al., 2022). Recently, it has also been reported that Ciliatoside A induced autophagy-lysosomal pathway degradation of HBc through activation of the AMPK-unc-51 like autophagy activating kinase 1 (ULK1) pathway and inhibition of mTOR, leading to significant suppression of both HBsAg expression and cccDNA transcription (Fang et al., 2023). Furthermore, Li et al. provided additional evidence that Asiatic acid promoted HBx protein degradation via the autophagy pathway, thereby reducing its binding to HBV cccDNA minichromatin. This process induced repressive epigenetic modifications and significantly suppressed cccDNA transcriptional activity, presenting a novel therapeutic approach for HBV treatment through HBx targeting (Li et al., 2024). As reported by Kirstgen et al., from the group of pentacyclic lupane-type betulin-derived triterpenoids, we identified a selective inhibitor of HBV entry that specifically blocked viral pre-S1 protein binding to NTCP while preserving NTCP’s bile acid transport function (Kirstgen et al., 2020). A research team designed and synthesized 32 novel conjugates through molecular hybridization of pentacyclic triterpenoids (glycyrrhetinic acid (GA), oleanolic acid, ursolic acid, and betulinic acid) with podophyllotoxin. Among these, four lead compounds (including BA-PPT3) specifically and competitively bound to the NTCP receptor’s 157–165 epitope, effectively blocking the HBV PreS1-NTCP interaction. This dual mechanism not only inhibited viral entry but also significantly reduced both HBsAg secretion and HBV DNA replication (Chen Y, et al., 2024).

##### 3.2.1.5 Other compounds

Beyond the major classes mentioned above, other types of natural compounds such as polysaccharides and saponins also demonstrate significant anti-HBV activities. Polysaccharide components showed unique advantages in anti-HBV research. In a study conducted by Wang et al., polysaccharide from Radix Isatidis activated JAK/STAT signaling pathway, dose-dependently reducing HBsAg/HBeAg levels and HBV DNA load in HepG2.2.15 cells, while promoting IFN-α production and suppressing negative regulators suppressor of cytokine signaling (SOCS), thereby exerting anti-HBV effects via an interferon-mimicking antiviral mechanism (Wang T, et al., 2020). Interestingly, it has been discovered that polysaccharides from Thais clavigera (Küster) exhibited dose-dependent anti-HBV activity in both HepG2.2.15 cells and HBV-transgenic mice by elevating serum IL-12/IFN-γ levels, with its high-dose group showing superior suppression of hepatic inflammation and viral markers compared to the clinical drug tenofovir alafenamide (Tang et al., 2021). In addition, Tang et al. suggested that total saponins extracted from Abrus cantoniensis Hance significantly suppressed HBV DNA replication and HBsAg/HBeAg secretion by modulating the phenylalanine/tyrosine metabolic pathway (Yao et al., 2020). Of note, it has been reported that 3,7-dimethoxytropolone and its ester derivatives exhibited selective anti-HBV activity (Schiavone et al., 2022), and the combination of schisandrin C and luteolin synergistically exerted anti-HBV effects by dual mechanisms: suppressing HBV replication (via downregulating the HNF4α/ERK pathway) and activating the cyclic GMP-AMP synthase (cGAS)-stimulator of interferon genes (STING) pathway in macrophages to promote IFN-β production (Wu et al., 2024).

#### 3.2.2 Herbal formulas

Recent advances have enhanced our understanding of the multi-target mechanisms underlying herbal formulas in HB treatment, with growing body of research employing integrated approaches that combine network pharmacology, molecular docking, multi-omics analyses, and *in vitro*/*in vivo* experimental validation. The TiaoGanYiPi formula could modulated 11 key target genes including Cyclin A2, ABL proto-oncogene 1 and Cyclin dependent kinase 4 to promote hepatocyte proliferation while suppressing hepatic inflammation (Cao et al., 2021). Long Chai Fang (containing core active ingredients such as corydaline and isorhamnetin) could inhibit viral DNA replication and reduce viral rebound after drug withdrawal by regulating 185 key targets and the phospholipid metabolism pathway (Xu et al., 2021). HuaganJiedu Decoction inhibited the levels of HBsAg, HBeAg and HBV DNA by activating the FOXO4/ERK/HNF4α signaling pathway and showed a synergistic effect when combined with entecavir (Tong et al., 2025). Le-Cao-Shi formula exerted multi-target therapeutic effects against HBV infection by modulating RAR-related orphan receptor A (RORA) and CDK2 to regulate cell cycle progression and inflammatory responses, while concurrently ameliorating gut microbiota dysbiosis. This study elucidates its integrative mechanism through “compound-target-microbiota” interactions (Zhao et al., 2022). Yinzhihuang granules could modulate 13 core targets (including CDK2 and tumor protein 53 (TP53)), demonstrating dual therapeutic effects: antiviral activity against HBV and prevention of HBV-related HCC progression (Zhang et al., 2020). Liuweiwuling Tablets selectively induced apoptosis in HBV-replicating hepatocytes by activating the PI3K-AKT, CASP3 and P53 pathways, demonstrating significant inhibitory effects against both wild-type and drug-resistant HBV strains (Ge et al., 2024). These findings validated the multi-target mechanisms of herbal formulas, demonstrating their ability to coordinately regulate cell cycle progression and immune responses through multi-component synergy.

### 3.3 Combination therapies

Recent advances in combination therapies have demonstrated groundbreaking potential for CHB treatment. Current therapeutic strategies can be categorized into three principal approaches: 1) multi-target combinations employing agents that act on distinct stages of the viral life cycle; 2) combined antiviral and immunomodulatory regimens; and 3) integrated Chinese-Western medicine approaches, particularly innovative combinations of herbal and synthetic pharmaceuticals.

#### 3.3.1 Multi-target design

Researchers have successfully combined bepirovirsen, an antisense oligonucleotide (ASO), with NAs and confirmed the absence of pharmacokinetic interactions between the two therapies, which established a foundation for their clinical combination in HBV therapy (Han et al., 2025). Clinical studies have demonstrated that the combination of JNJ-3989 (an siRNA therapeutic), JNJ-6379 (a capsid assembly modulator), and NAs significantly reduced HBsAg levels, lowered HBV DNA relapse rates, and exhibited favorable safety profiles in patients with CHB (Agarwal et al., 2024). These encouraging data suggested that pentacyclic triterpene-zidovudine conjugate emerged as a potent dual-target therapeutic, simultaneously inhibiting HBV polymerase and NTCP with 442-fold enhanced HBsAg suppression and an 87.8-fold improved therapeutic index, achieving synergistic antiviral efficacy through coordinated viral entry blockade and DNA replication suppression (Chen et al., 2025).

#### 3.3.2 Antiviral-immunomodulatory combination therapy

It is important to note that sequential therapy with bepirovirsen followed by PEG-IFNα-2a has demonstrated both safety and efficacy, reducing relapse rates while improving sustained HBsAg and HBV DNA clearance rates, particularly in patients with baseline HBsAg ≤3000IU/ml (Buti et al., 2025). A recent clinical trial demonstrated that selgantolimod (TLR8 agonist) combined with TAF presented good safety, induced immune responses, and produced a moderate decrease in HBsAg (though not achieving functional cure) in previously untreated CHB patients during 24 weeks of treatment, suggesting potential for further study of this combination therapy (Janssen et al., 2023). The clinical trial revealed that combination therapy with the siRNA therapeutic VIR-2218 and pegylated interferon-α2a (PEG-IFNα-2a) induced significant HBsAg reduction (up to 3.0 log10 IU/mL) in CHB patients. Notably, 11 patients (13.1%) achieved HBsAg clearance, with 6 cases (7.1%) maintaining sustained virologic response for 24 weeks post-treatment, all while demonstrating a manageable safety profile (Yuen et al., 2024). It is noteworthy that the novel immunomodulator AIC649 demonstrated synergistic antiviral effects with entecavir in the woodchuck model of CHB, exhibiting good safety without severe liver injury and supporting further development as a potential HBV therapeutic strategy (Korolowicz et al., 2021).

#### 3.3.3 Integrated Chinese-Western therapies

Integrated Chinese-Western therapies have achieved remarkable progress in CHB treatment, offering synergistic antiviral effects and potential pathways toward functional cure. A clinical study evaluating a 48-week regimen of adefovir dipivoxil combined with traditional Chinese medicine (Tiaogan Jianpi Hexue and Tiaogan Jiedu Huashi formulae) in HBeAg-positive CHB patients showed significantly enhanced HBeAg seroclearance rates without increased adverse events, demonstrating both the safety and superiority of this integrated Chinese-Western therapeutic approach (Li et al., 2020). Clinical evidence demonstrates that silymarin combined with standard antiviral therapy significantly reduces mortality in HBV-related cirrhotic patients while improving liver function parameters and comorbidity indices, providing a new option for comprehensive cirrhosis management (Huang et al., 2024). Interestingly, it has been discovered that electroacupuncture combined with tenofovir significantly enhanced anti-HBV efficacy by modulating the peroxisome proliferator-activated receptor (PPAR)-JAK/STAT pathway, resulting in a 5-fold greater decline in HBsAg levels compared to monotherapy while simultaneously improving intestinal barrier function, thereby offering a novel combination approach for achieving functional cure in HBV infection (Yang Y, et al., 2024).

## 4 Novel drug delivery systems

Significant progresses have been made in the research of novel drug delivery systems for HB treatment, primarily focusing on improving drug targeting, prolonging therapeutic duration, and enhancing treatment efficacy. Multiple studies have developed various innovative delivery platforms utilizing nanotechnology. More recently, a team developed a virus-trapping nanocage using DNA origami nanoshells, which was functionalized with HBV-specific monoclonal antibodies on its inner surface, achieving nearly 100-fold higher neutralization efficiency against HBV in cell culture compared to free antibodies (Willner et al., 2024). It had been reported by Guan’s group in 2024 that the GA-mediated intracellular breakable brucine, novel nano-drug delivery system, significantly enhanced brucine’s water solubility, stability, and hepatic targeting efficacy while reducing its toxicity, demonstrating potent anti-HBV activity in both *in vitro* and *in vivo* (Guan et al., 2024). In addition, Guan et al. successfully constructed a GA-modified polyethylene glycol-poly(lactic-co-glycolic acid) (PEG-PLGA) nano-drug delivery system co-loading syringopicroside and hydroxytyrosol, which formed uniform spherical nanoparticles (110.5 ± 3.18 nm) and significantly enhanced drug bioavailability through GA-mediated liver targeting, demonstrating superior anti-HBV efficacy over monotherapies in both *in vitro* and *in vivo* (Guan et al., 2023). Furthermore, Xu et al. successfully developed apolipoprotein A1-modified liver-targeting liposomes encapsulating baicalin, which enhanced the bioavailability and hepatic accumulation, effectively suppressing HBsAg, HBeAg, HBV RNA and DNA levels (Xu W, et al., 2022). Excitingly, research indicated that 1,2-Dioleoyl-3-trimethylammonium propane (DOTAP)-formulated cationic lipid-assisted nanoparticles (CLANs) efficiently targeted both plasmacytoid dendritic and conventional dendritic cells, and when combined with recombinant HBsAg, induced potent therapeutic immunity that achieved HBV clearance and ameliorated chronic liver inflammation in HBV carrier mice (Chen et al., 2023).

While NAs (e.g., entecavir, tenofovir) remain the cornerstone of HBV antiviral therapy, their clinical utility is limited by poor hepatic targeting and long-term toxicity. Recent advances in nanodelivery systems aim to optimize their therapeutic efficacy. It has been found that the GA and entecavir co-loaded albumin nanoparticle significantly enhanced entecavir accumulation in liver tissues by GA-mediated inhibition of hepatocyte efflux transporter activity (He et al., 2018). It is important to note that the sustained-release poly lactic-co-glycolic acid microspheres incorporating ETV in crystalline, amorphous and molecular states exhibited dissolution rate-controlled release kinetics *in vitro* and *in vivo*, providing an effective injectable long-acting formulation that prevented HBV viral rebound while reducing dosing frequency and improving patient compliance (Zhang et al., 2019). Moreover, the vitamin E-coated polymer hybrid nanoplatform with optimized lecithin-glyceryl monostearate lipid shells achieved high entecavir loading (80.47%) and liver-targeting particle size (188.66 nm), demonstrating sustained release over one week, enhanced macrophage uptake, and excellent serum stability (6 months at 4°C), thereby establishing a novel long-acting injectable platform combining dual advantages of polymeric nanoparticles and liposomes for improved HBV treatment (Hamdi et al., 2020). It is noteworthy that a novel antiviral delivery system based on reversible addition-fragmentation chain-transfer (RAFT) polymerization conjugated the TAF prodrug with hydrophilic polymers to form diblock copolymers (pTAF), incorporated them into polycaprolactone electrospun fiber scaffolds, and achieved sustained TAF release for up to two months, offering an innovative long-acting non-oral administration strategy to enhance HB treatment accessibility, reduce dosing frequency, and minimize side effects (Dart et al., 2022). Dong et al. developed a carrier-free metal-organic hybrid nanoassembly formed through self-assembly of tenofovir, an anti-viral agent and phosphorylated GA, an anti-inflammatory compound with zirconium cation, creating a high drug-loading nanostructure that prolonged blood circulation, enabled liver-targeted delivery, and achieved phosphatase-triggered drug release in HBV-infected hepatocytes, demonstrating dual synergistic therapeutic effects of antiviral activity and hepatoprotection in mouse models (Dong et al., 2022). Additionally, a study modified lamivudine into a lipophilic monophosphorylated prodrug, and this nanoformulation maintained effective drug concentration for 4 weeks and achieved sustained viral load reduction of approximately 1 log10 in HBV-infected humanized mice after a single intramuscular injection (75 mg/kg), with its 200–300 nm nanoparticles significantly enhancing liver targeting through hepatic macrophage-mediated delivery (Wang W, et al., 2020).

## 5 Conclusions and future prospects

While significant progress has been made in HBV therapeutics in recent years, substantial gaps remain in achieving WHO’s ambitious goal of eliminating HB by 2030. This review provides a comprehensive analysis of current advancements in HBV treatment, systematically examining breakthroughs in novel therapeutic targets, innovative drug development, combination strategies, and advanced drug delivery systems. Our work aims to offer researchers a holistic perspective on the current landscape, critically analyze existing therapeutic limitations, and explore viable pathways toward functional cure. The significance of this review lies in its integration of cutting-edge global research findings, identification of key research directions, and emphasis on translating basic research into clinical applications.

Although current NAs effectively suppress viral replication, they achieve functional cure rates below 10%, constrained by persistent cccDNA reservoirs, continuous antigen expression from integrated viral DNA, and profound immune tolerance in chronic infection. Notably, while novel direct-acting antivirals like CAMs and siRNA therapies demonstrate promising HBsAg reduction in clinical trials, questions remain regarding their ability to establish durable immune control. We contend that current research may overemphasize virological markers while neglecting the crucial aspect of host immune reconstitution, particularly in reversing T cell exhaustion and restoring effective HBV-specific immune responses. In drug development, although cccDNA-targeting gene editing technologies like CRISPR show laboratory success, their clinical translation faces major challenges including delivery efficiency, off-target effects, and long-term safety concerns. While combination therapies hold great promise, most current approaches merely combine drugs with different mechanisms rather than developing precisely coordinated immunomodulatory regimens. It is imperative to design potent combination strategies based on complementary and synergistic mechanisms, which can achieve dual objectives: directly attacking the virus while restoring robust host immune function to eliminate infected cells. More alarmingly, severe treatment inequity exists globally, with over 90% of HBV carriers in low-income countries lacking access to advanced therapies due to prohibitive costs.

Promisingly, natural products and integrated Chinese-Western therapies present a unique, complementary strategy for achieving HBV functional cure. Monomeric natural compounds (e.g., phenylpropanoids, flavonoids) offer distinct pharmacological advantages through their pleiotropic mechanisms, simultaneously targeting multiple viral life cycle stages (e.g., cccDNA transcription, NTCP entry) and modulating host immunity, which aligns with the need for rational combination strategies. Furthermore, clinical studies of herbal formulas combined with conventional nucleos(t)ide analogs have demonstrated enhanced serological response rates, underscoring their translational potential. However, clinical translation is hampered by significant challenges, including the poor bioavailability of monomeric compounds and the inherent complexity and compositional uncertainty of multi-herb formulations. Future efforts must therefore leverage advanced delivery systems (e.g., GA-modified nanoparticles) to improve pharmacokinetics, coupled with rigorous phytochemical characterization and well-designed clinical trials to standardize formulations and validate synergistic efficacy, thereby unlocking the full potential of these approaches within a modern therapeutic framework.

In the optimization of drug delivery systems, enhancing liver targeting and therapeutic duration has moved beyond an option to a necessity. Future research on delivery platforms must build upon this foundation. Systems like GA-modified nanoparticles (Guan et al., 2023, 2024) and A1-modified liposomes (Xu W, et al., 2022) demonstrated superior hepatocyte-specific delivery and enhanced bioavailability of encapsulated drugs (e.g., entecavir, baicalin). Their biocompatibility and targeting efficacy make them strong candidates for clinical translation, particularly for delivering small molecules and natural products. Furthermore, in resource-limited settings, the development of sustained-release formulations, such as microspheres and prodrug nanoformulations, that maintain effective drug concentrations for weeks or months could significantly improve treatment adherence. This is particularly critical for chronic HBV therapy, where unsupervised treatment interruption often leads to virologic rebound.

To translate current breakthroughs into clinical cures, future efforts must prioritize the following concrete and interconnected pathways: 1) deepen understanding of HBV immune evasion mechanisms; 2) develop cccDNA-specific clearance technologies that preserve genomic integrity; 3) establish more clinically relevant animal models; and 4) promote global treatment equity. Although functional cure has been achieved in some patients, transforming these successes into widely applicable clinical protocols requires overcoming numerous scientific and practical barriers. While the 2030 target may be overly optimistic, through sustained innovation and global collaboration, we remain confident that HB will ultimately be conquered. This endeavor demands not only scientific ingenuity but also concerted efforts from policymakers, pharmaceutical companies, and public health practitioners worldwide.
